# Clinical and radiographic outcomes of adjacent segment degeneration after short-segment fixation for lumbar vertebral fracture

**DOI:** 10.3389/fsurg.2026.1780109

**Published:** 2026-04-01

**Authors:** Jie Liu, Liang Wu, Ailiang Zhang, Xubin Qiu, Zhiwei Liu, Xiaofeng Yuan

**Affiliations:** 1 Department of Spine Surgery, The Third Affiliated Hospital of Soochow University, Changzhou, China; 2 Department of Orthopedics, Changzhou Maternal and Child Health Care Hospital, Changzhou Medical Center, Nanjing Medical University, Changzhou, China

**Keywords:** adjacent segment degeneration, lumbar, short-segment fixation, spine injuries, surgical outcome

## Abstract

**Objectives:**

This study aimed to examine the incidence and clinical implications of adjacent segment degeneration (ASD) following short-segment fixation for traumatic lumbar fractures and to compare the findings with the existing literature on degenerative spinal disease.

**Methods:**

This retrospective study included a cohort of 55 patients who underwent short-segment fixation for lumbar vertebral fractures. Radiographic ASD was evaluated using imaging modalities focusing on disc area reduction and facet joint degeneration. Clinical outcomes were quantified using the Visual Analog Scale (VAS) and Oswestry Disability Index (ODI) scores, with a mean follow-up duration of 14.02 months. Statistical analyses were conducted to compare outcomes between patients with and without ASD.

**Results:**

Radiographic ASD was observed in 36.4% of the patients. No statistically significant differences in the VAS or ODI scores were identified between the ASD and non-ASD groups throughout the follow-up period. The degenerative changes observed included a decreased disc area and erosion of the facet joints, which are consistent with the increased biomechanical stress experienced post-fusion. Importantly, degeneration of the distal segments was more prevalent in patients with traumatic injuries than in those with proximal segment degeneration, which is typically reported in cohorts with degenerative diseases.

**Conclusions:**

The mismatch between structural changes and symptoms indicates that ASD pathophysiology may vary between traumatic and degenerative conditions. Future research should involve larger samples and longer follow-up periods to better understand the long-term clinical effects.

## Introduction

Lumbar fractures are a prevalent category of spinal injuries. Surgical intervention is aimed at preserving neural function and restoring spinal stability. The standard range for long segment fixation typically encompasses two to three segments both superior and inferior to the injured vertebra. To mitigate the degeneration of unaffected segments, the objective of internal fixation in spinal injuries is to minimize the number of spinal levels involved in fixation or fusion. Consequently, short segment fixation has become the predominant surgical technique for unstable burst lumbar fractures, encompassing the injured vertebrae as well as the adjacent upper and lower vertebrae (1–3). Numerous studies have demonstrated that short segment fixation is effective in correcting kyphosis, restoring vertebral height, reducing canal encroachment, and improving patients' functional scores, such as Visual Analogue Scale (VAS) and Oswestry Disability Index (ODI) (4–9). In prior research examining complications following fixation, the primary concern was the failure of short-segment instrumentation (10, 11). In addition, adjacent segment degeneration (ASD) following lumbar spinal surgery remains a significant concern in spine surgery, particularly after short-segment fixation for lumbar vertebral fractures.

ASD is a comprehensive term that includes progressive narrowing of disc spaces, facet joint arthritis, and spinal instability at levels adjacent to fixed segments (12). This complication is primarily attributed to increased biomechanical stress on adjacent motion segments and altered load distribution patterns caused by spinal instrumentation, which collectively trigger a cascade of degenerative changes. Although advancements in surgical techniques and implant design have improved fracture stabilization outcomes, they have not substantially reduced the incidence of ASD. The current literature identifies multiple risk factors for ASD, including patient-specific variables, such as age, osteoporosis, and pre-existing degeneration, and surgical parameters, such as fusion length and sagittal alignment restoration (13). However, the pathophysiology remains poorly understood, with an ongoing debate about whether ASD arises predominantly from natural disease progression or iatrogenic biomechanical disruption. Notably, existing research predominantly focuses on ASD in degenerative conditions such as spondylolisthesis or spinal stenosis, while evidence specific to post-traumatic lumbar fractures remains scarce.

To address these knowledge gaps, this study aimed to analyze the clinical and radiographic outcomes of ASD following short-segment fixation of lumbar vertebral fractures. By integrating biomechanical principles, sagittal alignment metrics, and patient-reported outcomes, we sought to identify predictive factors for ASD and to refine surgical strategies to minimize its incidence. Our findings may provide targeted guidance for optimizing fracture management while mitigating debilitating postoperative complications.

## Methods

### Patient selection

The inclusion criteria were as follows: (1) single-segment fracture; (2) within two weeks of the injury; (3) Frankel Grade E, no neurologic deficits; (4) no spinal stenosis symptoms or low back pain; and (5) no previous lumbar surgery.

The exclusion criteria were as follows: (1) inability to tolerate surgery in the prone position; (2) blood coagulation dysfunction; (3) serious osteoporosis; (4) multi-segmental fractures; (5) the presence of other organ injury; (6) concurrent spinal cord compression or nerve injury; and (7) previous history of trauma or lumbar surgery.

### Radiological analysis and clinical outcome evaluation

**Figure 1:**
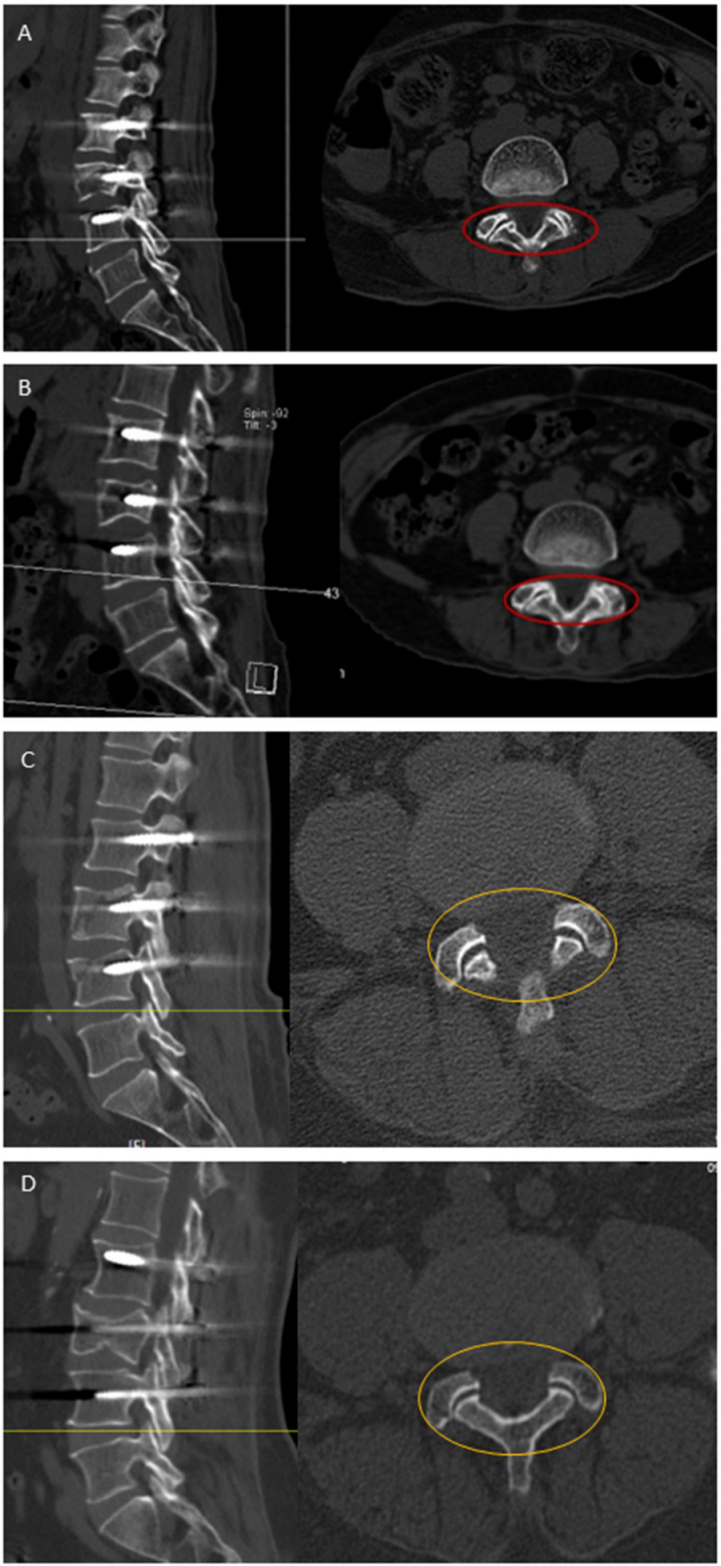
Radiographic change of bilateral facet joint degeneration in adjacent segments after short segment fixation. **(A,B)** Case 1, female, 61 years old, L3 fracture. A CT scan executed 1 week post-op **(A)** and 1 year post-op **(B)** clearly shows bilateral facet joint degeneration in adjacent segments. The red circle indicates a marked reduction or potential fusion of the joint space in the postoperative facet joint. **(C,D)** Case 2, male, 61 years old, L3 and L4 fracture. A CT scan executed 1 week post-op **(C)** and 17 months post-op **(D)** clearly shows bilateral facet joint degeneration in adjacent segments. The yellow circle indicates a marked reduction in joint space and the presence of bone hyperplasia in the postoperative facet joint.

The radiographic grading system of Weishaupt, D et al. was used to determine the degree of facet joint degeneration (14). On Magnetic resonance imaging(MR) and computed tomography (CT), the degree of facet joint degeneration was graded on a scale from 0 to 3 [grade 0: normal facet joint space (2 ± 4 mm width); grade 1: narrowing of the facet joint space (<2 mm) and/or small osteophytes and/or mild hypertrophy of the articular process; grade 2: narrowing of the facet joint space and/or moderate osteophytes and/or moderate hypertrophy of the articular process and/or mild subarticular bone erosions; grade 3: narrowing of the facet joint space and/or large osteophytes and/or severe hypertrophy of the articular process and/or severe subarticular bone erosions and/or subchondral cysts). Based on MR and CT images, degeneration of the upper and lower facet joints was defined as Weishaupt grades 2 and 3 (Figure 1).

**Figure 2:**
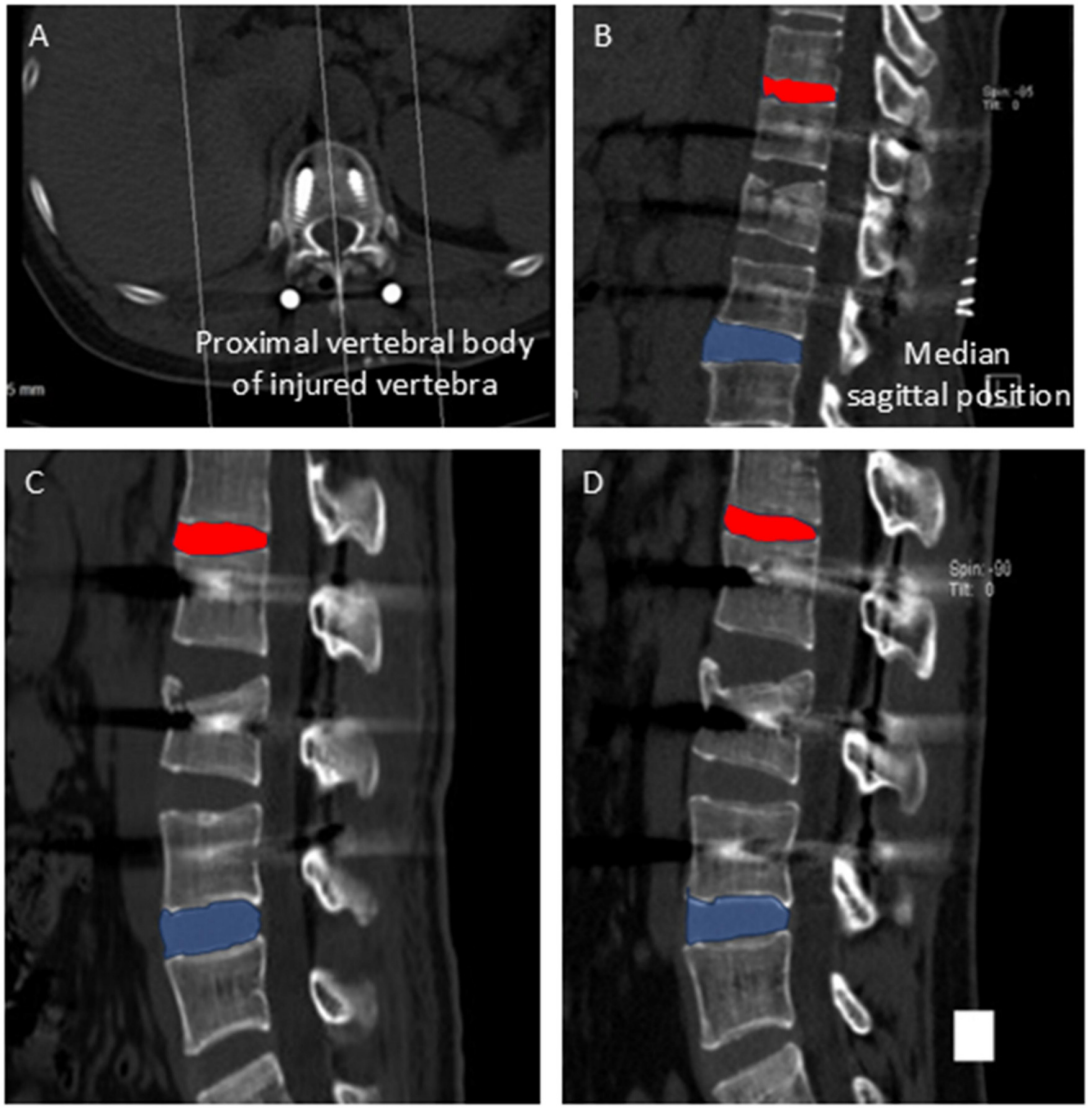
Radiographic change of intervertebral disc degeneration in adjacent segments after short segment fixation. **(A,B)** Selection of the measurement layer of intervertebral disc area: median sagittal position of the proximal vertebral body of the injured vertebra. Red area represents proximal intervertebral disc, and the blue area represents distal intervertebral disc. **(C,D)** Case 3, male, 25 years old, L3 fracture. A CT scan executed one week post-op **(C)** and fifteen months post-op **(D)** clearly shows reduction of sagittal cross-sectional area of intervertebral disc in adjacent segments after short segment fixation.

In our study, “proximal adjacent segment degeneration” refers to degenerative changes at the segment just above the fixed levels, while “distal ASD” refers to changes just below. For example, in an L1 fracture treated with fixation from T12-L2, T11-T12 is the proximal segment, and L2-L3 is the distal segment. This definition is consistently used in our analysis. The volumetric CT image dataset was reconstructed on a dedicated workstation (SESAN_RIS V1.0, Jiangsu, China) using built-in software to yield true sagittal images. The disc areas of the proximal or distal adjacent segments were measured at the median sagittal position (Figure 2). We chose three equally experienced senior chief surgeons to independently conduct lumbar spine measurements, with all results reviewed by the research team. Lumbar disc degeneration was confirmed if a measurable change in disc area between the immediate post-operative scan and the final follow-up was consistently identified by at least two of the three measurers and verified upon collective review by the research team.

At the time of the last follow-up, radiographic ASD was defined as having contain either one or both of the following changes: facet joint degeneration (Weishaupt grade 2 and 3) and lumbar disc degeneration.

The surgical segment, operation time, VAS score, and ODI score at different time points after surgery were recorded for all patients.

### Data analysis

Statistical analysis of the research data was conducted using SPSS 22.0. Baseline patient characteristics are presented as mea*n* ± standard deviation or absolute numbers and percentages. For within-group comparisons of the proximal and distal intervertebral disc area between the initial postoperative measurement and the final follow-up measurement, paired Student's t-tests were employed. An independent t test was used to test for differences in the means of radiological parameters and clinical outcome scores between patients with and without ASD. *P* < 0.05 is considered statistically significant.

## Results

### Study population

**Figure 3:**
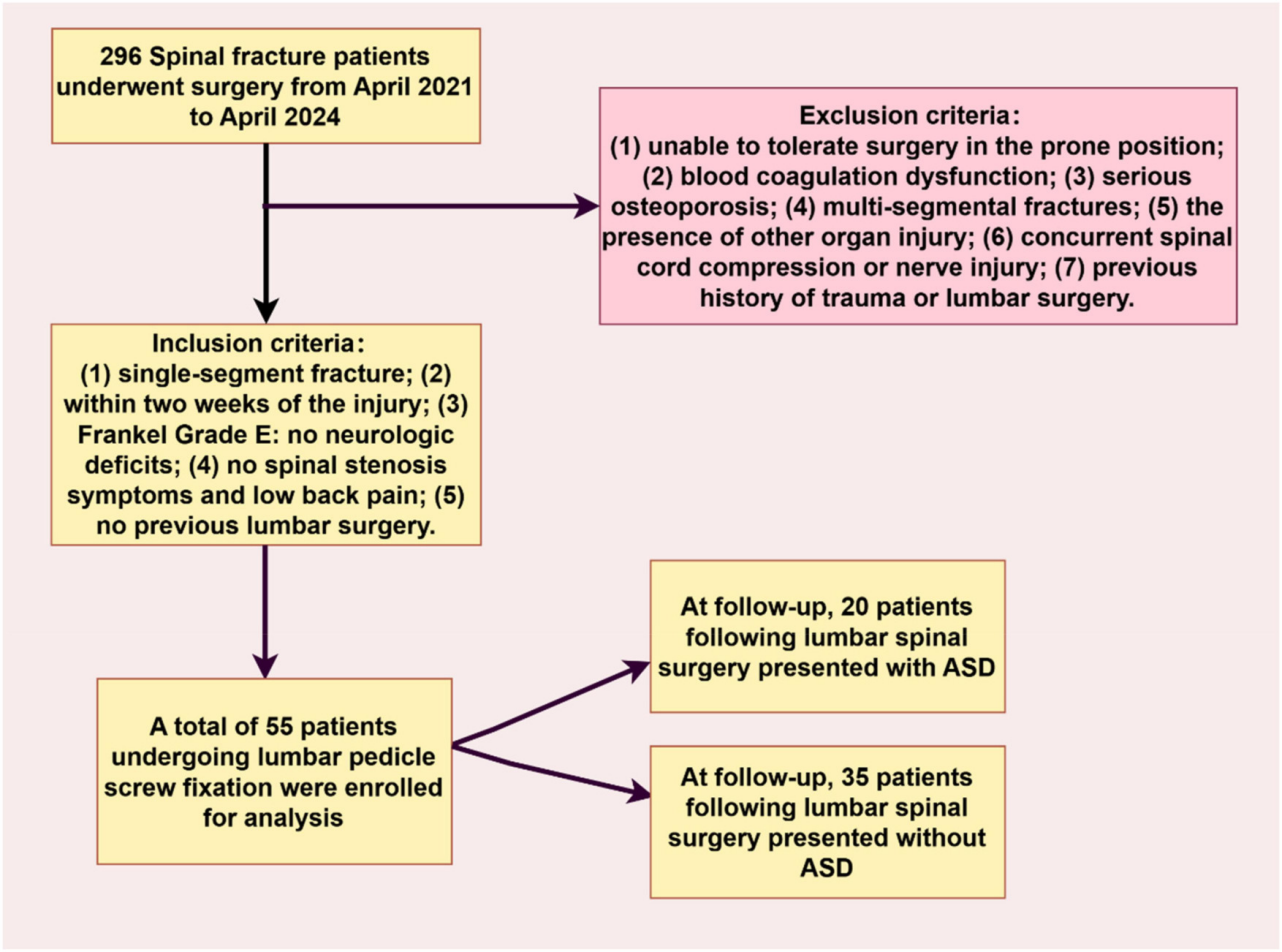
Flowchart of the participants included in the analysis.

The current study was approved by the Medical Ethics Committee of The Third Affiliated Hospital of Soochow University (ID: 2025-032), and each patient's consent was obtained for this study. A total of 296 patients with spinal fractures were enrolled in this study between April 2021 and April 2024. Accordingly, 241 participants with thoracic vertebral fractures, L5 fractures, two or more fractures, or spinal cord injury who needed decompression were excluded. Following the completion of this screening process, the study included 55 patients who underwent lumbar pedicle screw fixation with six screws placed in the fractured vertebra and the adjacent upper and lower vertebrae, which aimed to analyze the incidence or risk factors of ASD after lumbar fixation (Figure 3).

A total of 55 patients were enrolled in this study, including 34 males (61.8%) and 21 females (38.2%). The mean age was 50.07 ± 11.87 years (range: 24–67 years), and the mean BMI was 24.34 ± 2.69 kg/m^2^. Preoperative VAS and ODI scores were 5.96 ± 1.23 and 34.51 ± 5.86, respectively. Postoperative improvements were observed: VAS scores decreased to 1.78 ± 0.81 at 1 week and 0.56 ± 0.66 at the final follow-up, while ODI scores decreased to 19.07 ± 3.66 at 1 week and 10.80 ± 2.82 at the final follow-up. Fracture distribution included L1 (47.3%, *n* = 26), L2 (25.5%, *n* = 14), L3 (21.8%, *n* = 12), and L4 (5.5%, *n* = 3). The mean operative time was 1.43 ± 0.33 h, and the mean follow-up duration was 14.02 ± 2.63 months (Table 1).

**Table 1:** Demographic and clinical characteristics.

Parameters	*N* (%)/mean ± s
Age, years, mean ± SD	50.07 ± 11.87
Sex, *n* (%)	
Male	34 (61.8)
Female	21 (38.2)
BMI, kg/m^2^, mean ± SD	24.34 ± 2.69
VAS, mean ± SD	
Before operation	5.96 ± 1.23
1 week after operation	1.78 ± 0.81
Final follow-up	0.56 ± 0.66
ODI, mean ± SD	
Before operation	34.51 ± 5.86
1 week after operation	19.07 ± 3.66
Final follow-up	10.80 ± 2.82
Segment, *n* (%)	
L1	26 (47.3)
L2	14 (25.5)
L3	12 (21.8)
L4	3 (5.5)
Duration of operation, hour, mean ± SD	1.43 ± 0.33
Follow-up time, month, mean ± SD	14.02 ± 2.63
Proximal degeneration at final follow-up, *n* (%)	5 (9.1)
Distal degeneration at final follow-up, *n* (%)	19 (34.5)

SD, standard deviation; BMI, body mass index; VAS, visual analogue scale; ODI, oswestry disability index.

### Incidence of ASD and changes in intervertebral disc area after short segment fixation

As illustrated in Table 1, proximal degeneration was identified in 5 patients (9.1%), while distal degeneration was observed in 19 patients (34.5%) at the final follow-up. Paired Student's t-tests revealed statistically significant reductions in the intervertebral disc area for both proximal and distal segments at the final follow-up compared to initial postoperative measurements (proximal segment: 226.99 ± 64.84 mm^2^ vs. 205.51 ± 61.96 mm^2^, t = 7.444, *P* < 0.001; distal segment: 379.95 ± 81.95 mm^2^ vs. 323.14 ± 76.47 mm^2^, t = 9.191, *P* < 0.001) (Table 2). These findings indicate a notably high incidence of adjacent segment degeneration, suggesting that its occurrence is almost inevitable and warrants significant attention in clinical practice. It is also noteworthy that the probability of degeneration is greater in the distal segment than the proximal segment.

**Table 2:** Comparison of proximal and distal intervertebral disc area between initial postoperative and final follow-up measurements.

Parameters	Initial Postoperative	Final Follow-Up	t-value	*P*-value
Proximal Intervertebral Disc Area, mm^2^, mean ± SD	226.99 ± 64.84	205.51 ± 61.96	7.444	<0.001
Distal Intervertebral Disc Area, mm^2^, mean ± SD	379.95 ± 81.95	323.14 ± 76.47	9.191	<0.001

SD, standard deviation.

### Comparison of age, BMI, final follow-Up VAS, and ODI scores between non-degeneration and degeneration groups

Independent Student's t-tests indicated that there were no statistically significant differences between the non-degeneration group (*n* = 35) and the degeneration group (*n* = 20) with respect to age (48.80 ± 11.96 vs. 52.30 ± 11.68 years, t = −1.053, *P* = 0.297), BMI (24.30 ± 2.27 vs. 24.42 ± 3.38 kg/m^2^, t = −0.152, *P* = 0.880), final follow-up VAS scores (0.51 ± 0.66 vs. 0.65 ± 0.67, t = −0.730, *P* = 0.468), or final follow-up ODI scores (10.69 ± 2.70 vs. 11.00 ± 3.09, t = −0.394, *P* = 0.695) (Table 3). Our analysis revealed that the baseline characteristics of both groups were comparable, and their clinical outcomes within a follow-up period of less than two years were similar.

**Table 3:** Patient characteristics between non-ASD and ASD groups.

Parameters	Non-ASD group (*n* = 35)	ASD group (*n* = 20)	t-value	*P*-value
Age, years, mean ± SD	48.80 ± 11.96	52.30 ± 11.68	−1.053	0.297
BMI, kg/m^2^, mean ± SD	24.30 ± 2.27	24.42 ± 3.38	−0.152	0.880
VAS at latest follow-up, mean ± SD	0.51 ± 0.66	0.65 ± 0.67	−0.730	0.468
ODI at latest follow-up, mean ± SD	10.69 ± 2.70	11.00 ± 3.09	−0.394	0.695

BMI, body mass index; VAS, visual analogue scale; ODI, oswestry disability index; SD, standard deviation.

## Discussion

Adjacent segment degeneration remains a significant complication of short-segment fixation for lumbar vertebral fractures. In our study, 36.4% of the patients developed radiographic evidence of ASD over a mean follow-up period of 14.02 months, with the more commonly affected level being the distal segment compared to the proximal segment. Previous reports have indicated an absence of correlation between imaging-detected degeneration and clinical symptoms (15–17). This suggests that despite the presence of clear evidence of degeneration on imaging, patients may not experience any associated discomfort. Our results show that even if ASD occurs in the short term after surgery, the VAS and ODI scores will not differ significantly from those of normal postoperative patients. These findings are consistent with previous reports.

ASD development is influenced by various biomechanical and surgical factors (15, 18, 19). Our study demonstrated a significant reduction in the disc area between adjacent segments, which may be attributed to a notable increase in intervertebral disc pressure and segmental motion (20–23). These findings align with those of previous reports on the biomechanical effects of lumbar spine surgery on adjacent segments (22). The facet joints endure significant forces to protect lumbar discs from shear loads and resist rotational forces (22). Posterior internal fixation may change spine load transfer and movement, potentially increasing stress on nearby small joints, highlighting the biomechanical importance of lumbar facet joints and their capsules. Therefore, we observed degeneration of the adjacent segment facet joints, characterized by narrowing of the joint space and erosion of the subchondral bone (14).

Upon comparing our results with the existing literature on degenerative spinal diseases, several intriguing findings emerged. Historically, lumbar fusion surgery for lumbar degenerative diseases has been associated with a higher likelihood of proximal adjacent segment degeneration (24). However, our study indicates that internal fixation for traumatic lumbar fractures may result in increased degeneration of distal adjacent segments. We posit that lumbar degenerative disease predominantly affects the L4/5 and L5/S1 segments. Given that the activity of the distal lumbosacral segment is diminished following fusion surgery, the proximal adjacent segment becomes the primary site of stress concentration and compensatory activity increase, thereby making proximal degeneration more probable post-fusion surgery. The thoracolumbar junction (TLJ) is the junctional region between the thoracic and lumbar vertebrae (25). This region represents a pivotal transition zone in terms of spinal biomechanics and morphology, where the thoracic spine's kyphotic curvature converges with the lumbar spine's lordotic curvature, thereby generating considerable shear forces and axial loads (26, 27). The thoracolumbar junction functions as a transitional zone between the relatively rigid thoracic spine and the highly mobile lumbar spine, inherently acting as a stress concentration point. While short-segment posterior fixation restores the alignment of the spinal column, it also increases the rigidity of the fixed segments, resulting in a compensatory distal shift of the center of motion (L3-S1). Trauma patients typically do not have a prolonged history of degeneration prior to surgery, and thus, their lumbar lordosis is generally pronounced. Postoperatively, to compensate for the mild kyphosis that may develop in the fixed segments, the distal segments (particularly L4/5 and L5/S1) often experience increased facet joint loading to maintain sagittal balance, thereby accelerating distal degeneration. This biomechanical complexity is proposed as a primary factor contributing to the heightened susceptibility of the adjacent segment to complications following internal fixation surgery for lumbar fractures (28). Furthermore, this result underscores the necessity of considering that the pathophysiology of ASD post-trauma and fusion may involve distinct mechanisms, warranting separate discussion and analysis.

This study had several limitations that warrant acknowledgment. It is particularly significant to note that all fixation techniques utilized in our study incorporated a 6-screw fixation approach. We hypothesize that the use of different fixation techniques may result in varying degrees of degenerative outcomes. Consequently, future research will involve a comparative analysis of the degeneration outcomes of adjacent segments following the application of diverse fracture fixation methods. The retrospective design may have introduced specific biases, and the limited sample size of 55 patients constrains the generalizability of the findings. Given the specific needs of patients, individuals with fractures typically undergo removal of internal fixation devices promptly after fracture healing. However, the follow-up period, limited to a maximum of two years, may be inadequate for comprehensively capturing the occurrence patterns and clinical characteristics of ASD over the medium to long term. For instance, our study found no statistically significant difference in the VAS and ODI scores between degenerated and non-degenerated patients over the two-year follow-up period. However, this does not preclude the possibility of differences in clinical symptoms that emerge over longer follow-up periods. This finding highlights the need to enhance the follow-up questionnaire to extend the observation period, thereby increasing the reliability of our conclusions. Comprehensive long-term surveillance is essential for a thorough understanding of the natural history and clinical implications of ASD. Given that ASD is a time-dependent condition, an extended follow-up duration is necessary to more accurately ascertain its true incidence and long-term clinical significance. Future studies should incorporate larger sample sizes and extended follow-up periods to address these limitations more effectively. Another major concern is that the radiographic characteristics of ASD vary and encompass intervertebral disc degeneration, facet joint degeneration, dynamic instability, Modic changes of the endplate (types I-III), new-onset spinal stenosis, and ligament hypertrophy. This study primarily focused on the prevalent manifestations, specifically intervertebral disc and joint degeneration. Consequently, there is a paucity of data regarding adjacent segment stability, endplate alterations, and changes in the spinal canal adjacent segment. In future research, we intend to enhance the sample size and broaden the scope of data collection to incorporate more comprehensive imaging findings.

## Conclusions

In conclusion, this study underscores that ASD is a prevalent complication following short-segment fixation for lumbar fractures, with a higher incidence observed in the distal segments than in the proximal segments. Although short-term clinical outcomes, as measured by VAS and ODI, did not show significant differences between patients with ASD and those without ASD, the long-term effects remain uncertain due to the limited duration of follow-up. The development of ASD is likely influenced by biomechanical stress and altered segmental motion. Importantly, post-traumatic ASD may differ from ASD associated with degenerative disease, highlighting the necessity for further research involving larger cohorts and extended follow-up periods to elucidate disease progression and optimize surgical strategies.

## Data Availability

The original contributions presented in the study are included in the article/Supplementary Material, further inquiries can be directed to the corresponding author/s.

## References

[1] Jindal R Jasani V Sandal D Garg SK. Current status of short segment fixation in thoracolumbar spine injuries. J Clin Orthop Trauma. (2020) 11(5):770–7. 10.1016/j.jcot.2020.06.008 32879564 PMC7452221

[2] Gelb D Ludwig S Karp JE Chung EH Werner C Kim T Successful treatment of thoracolumbar fractures with short-segment pedicle instrumentation. J Spinal Disord Tech. (2010) 23(5):293–301. 10.1097/BSD.0b013e3181af20b6 20606547

[3] Mahar A Kim C Wedemeyer M Mitsunaga L Odell T Johnson B Short-segment fixation of lumbar burst fractures using pedicle fixation at the level of the fracture. Spine (Phila Pa 1976). (2007) 32(14):1503–7. 10.1097/BRS.0b013e318067dd24 17572619

[4] Altay M Ozkurt B Aktekin CN Ozturk AM Dogan O Tabak AY. Treatment of unstable thoracolumbar junction burst fractures with short- or long-segment posterior fixation in magerl type a fractures. Eur Spine J. (2007) 16(8):1145–55. 10.1007/s00586-007-0310-5 17252216 PMC2200786

[5] Wang XY Dai LY Xu HZ Chi YL. Kyphosis recurrence after posterior short-segment fixation in thoracolumbar burst fractures. J Neurosurg Spine. (2008) 8(3):246–54. 10.3171/SPI/2008/8/3/246 18312076

[6] Lakshmanan P Jones A Mehta J Ahuja S Davies PR Howes JP. Recurrence of kyphosis and its functional implications after surgical stabilization of dorsolumbar unstable burst fractures. Spine J. (2009) 9(12):1003–9. 10.1016/j.spinee.2009.08.457 19819190

[7] Sjostrom L Karlstrom G Pech P Rauschning W. Indirect spinal canal decompression in burst fractures treated with pedicle screw instrumentation. Spine (Phila Pa 1976). (1996) 21(1):113–23. 10.1097/00007632-199601010-00026 9122751

[8] Gertzbein SD Crowe PJ Fazl M Schwartz M Rowed D. Canal clearance in burst fractures using the AO internal fixator. Spine (Phila Pa 1976). (1992) 17(5):558–60. 10.1097/00007632-199205000-00013 1621155

[9] Arlet V Orndorff DG Jagannathan J Dumont A. Reverse and pseudoreverse cortical sign in thoracolumbar burst fracture: radiologic description and distinction–a propos of three cases. Eur Spine J. (2009) 18(2):282–7. 10.1007/s00586-008-0848-x 19082845 PMC2899345

[10] McCormack T Karaikovic E Gaines RW. The load sharing classification of spine fractures. Spine (Phila Pa 1976). (1994) 19(15):1741–4. 10.1097/00007632-199408000-00014 7973969

[11] Mc Lain RF Sparling E Benson DR. Early failure of short-segment pedicle instrumentation for thoracolumbar fractures. A preliminary report. J Bone Joint Surg Am. (1993) 75(2):162–7. 10.2106/00004623-199302000-00002 8423176

[12] Virk SS Niedermeier S Yu E Khan SN. Adjacent segment disease. Orthopedics. (2014) 37(8):547–55. 10.3928/01477447-20140728-08 25102498

[13] Hashimoto K Aizawa T Kanno H Itoi E. Adjacent segment degeneration after fusion spinal surgery-a systematic review. Int Orthop. (2019) 43(4):987–93. 10.1007/s00264-018-4241-z 30470865

[14] Weishaupt D Zanetti M Boos N Hodler J. MR Imaging and CT in osteoarthritis of the lumbar facet joints. Skeletal Radiol. (1999) 28(4):215–9. 10.1007/s002560050503 10384992

[15] Rahm MD Hall BB. Adjacent-segment degeneration after lumbar fusion with instrumentation: a retrospective study. J Spinal Disord. (1996) 9(5):392–400. 10.1097/00002517-199610000-00005 8938607

[16] Booth KC Bridwell KH Eisenberg BA Baldus CR Lenke LG. Minimum 5-year results of degenerative spondylolisthesis treated with decompression and instrumented posterior fusion. Spine (Phila Pa 1976). (1999) 24(16):1721–7. 10.1097/00007632-199908150-00014 10472107

[17] Ghiselli G Wang JC Bhatia NN Hsu WK Dawson EG. Adjacent segment degeneration in the lumbar spine. J Bone Joint Surg Am. (2004) 86(7):1497–503. 10.2106/00004623-200407000-00020 15252099

[18] Boden SD McCowin PR Davis DO Dina TS Mark AS Wiesel S. Abnormal magnetic-resonance scans of the cervical spine in asymptomatic subjects. A prospective investigation. J Bone Joint Surg Am. (1990) 72(8):1178–84. 10.2106/00004623-199072080-00008 2398088

[19] Yang SW Langrana NA Lee CK. Biomechanics of lumbosacral spinal fusion in combined compression-torsion loads. Spine (Phila Pa 1976). (1986) 11(9):937–41. 10.1097/00007632-198611000-00014 3824071

[20] Kieffer SA Stadlan EM Mohandas A Peterson HO. Discographic-anatomical correlation of developmental changes with age in the intervertebral disc. Acta Radiol Diagn (Stockh). (1969) 9:733–9. 4909296

[21] Modic MT Masaryk TJ Ross JS Carter JR. Imaging of degenerative disk disease. Radiology. (1988) 168(1):177–86. 10.1148/radiology.168.1.3289089 3289089

[22] Leone A Guglielmi G Cassar-Pullicino VN Bonomo L. Lumbar intervertebral instability: a review. Radiology. (2007) 245(1):62–77. 10.1148/radiol.2451051359 17885181

[23] Natarajan RN Andersson GB. Lumbar disc degeneration is an equally important risk factor as lumbar fusion for causing adjacent segment disc disease. J Orthop Res. (2017) 35(1):123–30. 10.1002/jor.23283 27152925

[24] Soh J Lee JC Shin BJ. Analysis of risk factors for adjacent segment degeneration occurring more than 5 years after fusion with pedicle screw fixation for degenerative lumbar spine. Asian Spine J. (2013) 7(4):273–81. 10.4184/asj.2013.7.4.273 24353843 PMC3863652

[25] Liu CJ Zhu ZQ Wang KF Duan S Xu S Liu HY. Radiological analysis of thoracolumbar junctional degenerative kyphosis in patients with lumbar degenerative kyphosis. Chin Med J (Engl). (2017) 130(21):2535–40. 10.4103/0366-6999.217090 29067951 PMC5678250

[26] Cho KJ Suk SI Park SR Kim JH Jung JH. Selection of proximal fusion level for adult degenerative lumbar scoliosis. Eur Spine J. (2013) 22(2):394–401. 10.1007/s00586-012-2527-1 23064878 PMC3555634

[27] Moon HJ Bridwell KH Theologis AA Kelly MP Lertudomphonwanit T Kim HJ Thoracolumbar junction orientation: its impact on thoracic kyphosis and sagittal alignment in both asymptomatic volunteers and symptomatic patients. Eur Spine J. (2019) 28(9):1937–47. 10.1007/s00586-019-06078-y 31342155

[28] Passias PG Wang S Kozanek M Xia Q Li W Grottkau B Segmental lumbar rotation in patients with discogenic low back pain during functional weight-bearing activities. J Bone Joint Surg Am. (2011) 93(1):29–37. 10.2106/JBJS.I.01348 21209266 PMC3004094

